# A chest tube may not necessary in children thoracoscopic lobectomy

**DOI:** 10.1097/MD.0000000000015857

**Published:** 2019-06-28

**Authors:** Kaisheng Cheng, Miao Yuan, Chang Xu, Gang Yang, Min Liu

**Affiliations:** Department of pediatric surgery, West China hospital, Sichuan University, China.

**Keywords:** chest tube, meticulous resection, pediatric lung resections, postoperative management, thoracoscopic surgery

## Abstract

Drainage of the thorax postoperatively using chest tubes is a standard procedure in thoracic surgery. However, chest tubes can induce pain and immobilization, increase risk of infection, deteriorate the ventilation capacity, and increase difficulty of postoperative management, particularly in children. This study aimed to investigate the safety and effect of excluding chest tubes after performing thoracoscopic lobectomy in selected children.

A retrospective review of medical records was performed in West China Hospital of Sichuan University from January 2014 to June 2018. Patients who underwent thoracoscopic lobectomy without chest tubes were recorded. Patients with accompanying severe pulmonary infection, extensive thoracic adhesions, or undeveloped interlobar fissure were excluded.

In total, 246 patients underwent thoracoscopic lobectomy without a chest tube, and none required chest drain insertion or reintervention during hospitalization and follow-up at 90 days postoperatively. Among them, 2 (0.81%) patients developed a delayed pneumothorax which was found after being discharged, and resolved spontaneously in 2 weeks. No hemothorax, atelectasis, and bronchial fistula were found. Furthermore, 202 (82.1%) patients developed subcutaneous emphysema, which was asymptomatic and spontaneously resolved within 3 to 7 days. The length of postoperative hospital stay was 2 days; patients were discharged in the 3rd day postoperatively. Patients could recover to free mobilization and resume regular diet at 6 hours postoperatively. All patients were followed up for at least 3 months; no other complications were found, and all patients recovered well.

This study showed that chest tube placement in selected patients may be unnecessary in children undergoing thoracoscopic lobectomy. The minimally invasive procedure and meticulous resection have been the preconditions of this procedure, which may contribute to a rapid recovery and can avoid the chest tube-related complications effectively.

## 1 Introduction

The greatest concern after thoracic surgery is to monitor whether it would develop into a potentially life-threatening tension pneumothorax because of air leakage or a progressive hemothorax. Draining fluid as well as air and avoiding the accumulation of air, blood, and percolate in the pleural space by chest tube placement are standard procedures after thoracic surgery.^[1]^ However, chest tubes can induce morbidities such as pain and immobilization, increase the risk of infection, deteriorate the ventilation capacity,^[2–5]^ and increase difficulty of postoperative management, particularly in children.^[6]^ They not only cause discomfort in children, but also prolong the length of hospital stay and increase expenses.^[6]^ Therefore, in recent years, an increasing number of studies focused on problems regarding chest tube placement after thoracic surgeries.^[7]^

With the advances in the thoracic minimally invasive surgery and the generalization of the concept of precise operation, surgeons have paid more attention to elaborate manipulation and meticulous resection that made minimally invasive operation have the following advantages: less traumatic for patients, less effusion and more efficient. Therefore, in recent years, thoracoscopic operation had also attempted to reduce the rate of postoperative events requiring chest drainage.^[7,8]^ Okur et al and Gómez-Caro et al found that using a single tube is more effective than using 2 tubes, and they decreased the amount of chest tube drainage.^[2,9]^ Younes et al and Bjerregaard et al had attempted to increase air leakages detected intraoperatively to allow early removal of chest tubes in the postoperative course, and they had effectively reduced the hospitalization times and acquired a low post-operative complication rate.^[10–12]^ At the same time, other authors reported that by using fibrin glue or constantly amending the selection criteria of patients, they can make exclusion of chest tubes after pulmonary wedge resection a safe and feasible procedure, and found no increase in morbidity and mortality.^[13–17]^ Meanwhile, Ueda et al and Murakami et al had reported that they successfully avoided leaving chest tubes during major lung resection through the refined strategy for pneumostasis.^[18,19]^ With regard to children undergoing thoracoscopic operation, Todd had demonstrated that leaving no chest tubes (NCTs) after some no- lung manipulation (ductus arteriosus ligation, congenital diaphragmatic repair, among others) or tiny tissue dissection (lung biopsy) was feasible and allowed for a much more tolerable postoperative course in most children.^[6]^ However, considering the difficulty of dealing with children undergoing thoracoscopic anatomical lobectomy, to date, chest tube placement is a regular procedure, and the chest tube placement time should be at least 1.3 days.^[20]^ Currently, there are no reports onNCT placement after thoracoscopic lobectomy in children.

In our previous clinical observation of patients placed with chest tubes after thoracoscopic lobectomy, the majority of them were found with no obvious air leak and pleural effusion; hence, we gradually shortened the chest tube placement time and; until 2014, we began to exclude the use of postoperative chest tube in some selected patients.^[21]^ In this study, we reviewed the experience in NCT placement after thoracoscopic lobectomy in children in our hospital and evaluated the safety and effectiveness of this approach.

## 2 Methods

A retrospective review of medical records was performed on children undergoing thoracoscopic lobectomy in West China Hospital of Sichuan University from 2014 to 2018. Patients who underwent thoracoscopic lobectomy without chest tube placement were recorded; the other patients who did not meet the criteria were excluded. This study was approved by the Medical Ethics Committee of West China Hospital. This retrospective study was approved by the ethics committees of West China Hospital of Sichuan University (No.317, March 4, 2014). This retrospective study used a database from which the patients’ identification information had been removed.

### 2.1 Patient selection

All selected patients were diagnosed with congenital lung malformation by thin-section computed tomography preoperatively. All patients selected in our study underwent thoracoscopic anatomical lobectomy. Table 1 shows the inclusion criteria.

**Table 1:**
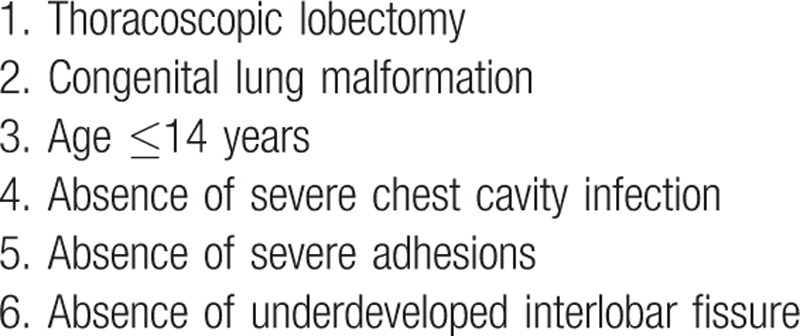
Inclusion criteria for postoperative chest drain omission.

### 2.2 Operative management

Patients underwent surgery under general anesthesia with single-lung ventilation using double or single lumen intubation with bronchial blockade or 2-lung ventilation, using artificial pneumothorax (5–10 mmHg, 1 L/min CO_2_ flow). The procedure was performed in a bimanual fashion. The surgeon used 2 instruments through 2 ports, whereas the camera was controlled by the assistant through the third port placed in the middle. Vessels, fissures, and bronchi were divided and sealed one-by-one with a hemolock, cautery hook, and Enseal (Ethicon Endo-Surgery, Inc., Cincinnati, OH) or LigaSure or endostapler, (Covidien, CT). The details were described in our previous study.^[21]^

### 2.3 Postoperative management

All patients were initially observed in a post-anesthesia care unit (PACU) for 2 to 3 hours by trained PACU nurses. Standard observations included heart rate, blood pressure, respiratory rate, temperature, oxygenation, and subcutaneous emphysema after admission to a standard general surgical ward. A low-dose chest computed tomography scan was obtained within the first 24 hours postoperatively to detect whether tension pneumothorax or pleural effusion developed, or examine whether atelectasis or bronchial fistula existed, or evaluate whether there were lesion residues. Approximately 90 days postoperatively, the patients were followed up in the outpatient clinic with clinical and radiological postoperative control. Postoperative complications were recorded both upon discharge and in the outpatient clinic during the 90-day follow-up for those patients in our hospital.

### 2.4 Statistical analysis

Continuous variables were presented as mean ± standard deviation (SD) or as median (25th–75th percentile) depending on normality. Categorical variables are presented as number (percentage). All statistical analyses have been performed using SPSS 22.0 (IBM SPSS Statistics, Chicago, IL).

## 3 Results

**Figure 1:**
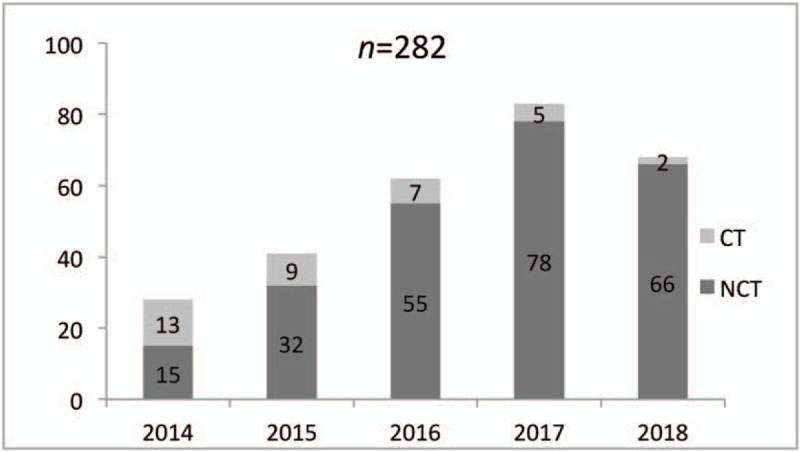
Since 2014, 282 thoracoscopic lobectomies have been completed by performing no-chest tube placement procedure. The proportion of no-chest tube placement had increased through the years.

From July 2014to July 2018, according to the criteria, 246 patients were selected to undergo thoracoscopic lobectomy with NCT placement. The case statistics are shown in Figure 1. Since 2014, the use of chest tube began to be excluded in postoperative management, and 15 (53.7%) patients had 1 chest tube placed and in 13 (46.3%) patients we avoided chest tube placement in the first year. The proportion of patients with NCT placement had increased over years, and 41 patients had undergone thoracoscopic lobectomy in 2015,with 32 (78.0%)of them avoiding chest tube placement. In 2016, 62 patients underwent thoracoscopic lobectomy, with 55 (88.7%) them avoiding chest tube placement. Eighty-three patients had undergone thoracoscopic lobectomy, with 78 (93.9%) avoiding chest tube placement in 2017. Until 2018, 68 patients had accomplished children thoracic lobectomy, and 66 (97%) did not have chest tube placement postoperatively in this year.

The postoperative complications are summarized in Table 2. Two patients (0.81%) developed symptomatic pneumothorax after being discharged and pneumothorax resolved spontaneously within 2 weeks, and neither required chest drain insertion or reintervention. No patient had hemothorax, atelectasis, or bronchial fistula in clinical or radiological control both upon discharge and in the follow-up. Furthermore, 202 patients (82.1%) developed a minor complication, subcutaneous emphysema, which was resorbed spontaneously in 3 to 7days, and 76 patients (30.9%) developed fever postoperatively, with temperature <38.5°C. None of those patients developed progressive emphysema, and those patients were asymptomatic and were discharged in 2 days.

**Table 2:**
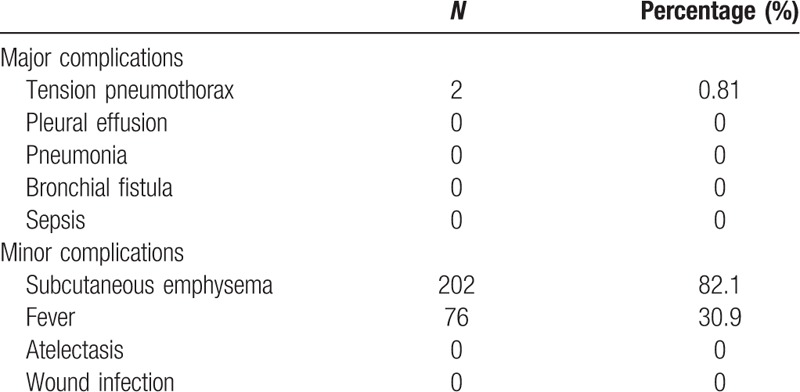
Postoperative Complications.

The operative outcome is summarized in Table 3. The mean age was 8.8 months, ranging from 3 to 20 months; there were 118 males and 128 female patients. The mean operative time was 48.2 minutes, ranging from 29 to 61 minutes. No patient developed pulmonary infection or atelectasis caused by chest tube. Patients could recover to free mobilization and resume regular diet in 6 hours after operation. The routine postoperative hospital stay was 2 days; all patients were discharged on the 3rd postoperatively day.

**Table 3:**
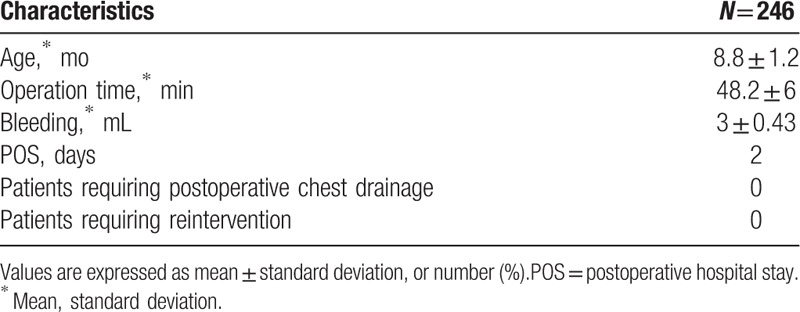
Operative outcome.

## 4 Discussion

In this study, 246 children successfully underwent thoracoscopic lobectomy with NCT placement and were followed up at least 90 days postoperatively. No patient developed potentially life-threatening tension pneumothorax and a progressive hemothorax. Only 2 patients (0.81%) developed a major symptomatic pneumothorax during the follow-up. They recovered well during the hospitalization, but both of them developed infectious mononucleosis within 1 week, with the symptoms of fever and drastic cough with a clinical and radiological pneumothorax, 1 week after being discharged. We speculated that it may be the continuous drastic cough, which led to air leaking of the incisional edge lung tissue rather than a bronchial fistula. After 2 weeks of medical conservative treatment, the pneumothorax did not progress and resolved spontaneously. All the other patients were rehabilitated well, no major complication occurred and none required chest drain insertion or reintervention during their hospitalization and in the 90-day follow-up period. Compared with previous studies, their data showed the following: proportion of patients with infection was 2.2%, postoperative atelectasis,^[22]^ and postoperatively hospital stay was 3 days.^[23,24]^ In our study, no patient developed pulmonary or thoracic cavity infection during hospitalization, and the routine postoperative hospital stay routinely was 2 days since we performed this procedure. In our study, we have decreased the risk of infection, postoperative atelectasis and postoperatively hospital stay, but increased the risk of subcutaneous emphysema. In this study, we also found that asymptomatic subcutaneous emphysema occurred in 202 patients (82.1%). We closely monitored the progression of subcutaneous emphysema. Nearly all subcutaneous emphysema resolved spontaneously within 3 to 7days. We conjectured that along with the expansion of the remnant lung tissue, the residual CO_2_ we infused intraoperatively may be extruded into the subcutaneous tissue space. Furthermore, 76 patients (30.9%) developed fever postoperatively, and their temperature was <38.5°C. They had no symptoms of cough or dehydration. Most of them have spontaneously recovered in 1 or 2 days, which may be because of the postoperative absorbing process, without chest tubes placement, a small amount of exudation is absorbed by the body postoperatively which may lead to fever of short duration. Durell et al have reported that nearly 1 in 4 patients with asymptomatic congenital lung malformation showed signs of subclinical infection,^[25]^ In this study, we have observed intraoperatively that many patients had infectious lesions; hence, we administered antibiotics for 1–2 days in all patients postoperatively to prevent this kind of subclinical infection. Meanwhile, without the use of chest tubes, no postoperative atelectasis occurred in these patients, and they were able to move freely and tolerate regular diet 6 hours after anesthesia recovery. Our data showed that NCT placement after children thoracoscopy lobectomy is safe for selected patients and has a low incidence of complications. A low-dose chest computed tomography was performed within 24 hours after operation to detect the occurrence of serious complications and to assess the presence of residual lesions. Although computed tomography radiation is considered to be associated with some malignant cancer,^[26]^ there were reports that some of these ionizing radiation-associated cancer cases may be preventable through dose optimization of and enhanced justification for diagnostic examinations.^[27,28]^

**Figure 2:**
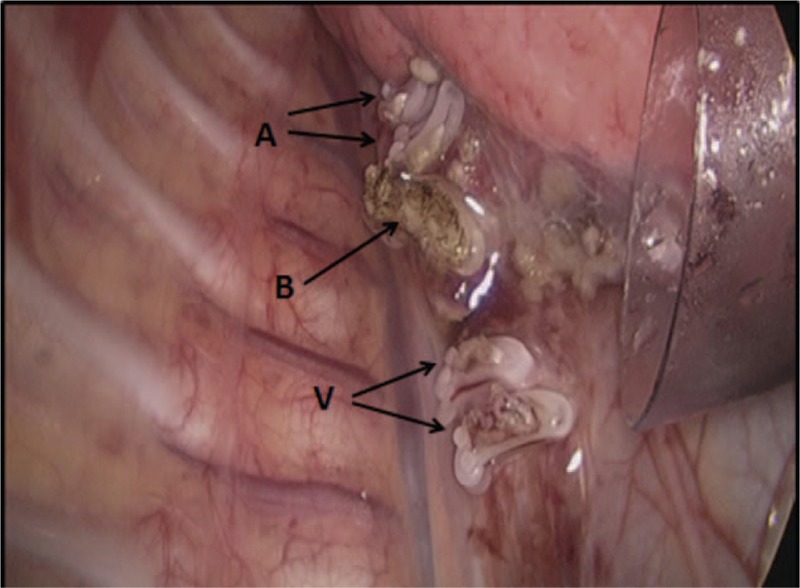
The pulmonary hilum structure at the end of the surgery. A, B, and V represent pulmonary artery, bronchus, pulmonary vein stumps, respectively.

The development of the thoracic minimally invasive surgery technique, the advances of the operating instruments and experienced surgeons who are able to proficiently master the precise operation are the significant preconditions of this procedure. In this study, the results showed that, from 2014 to 2018, the proportion of the patients without chest tube placement has increased gradually. Yamataka et al reported that these surgical skills have been the crucial point of successful lobectomy in children.^[29]^ Thoracoscopic minimally invasive surgery when adopting a meticulous technique is a reliable treatment of pulmonary diseases with low complication rate.^[30]^ In our previous study, we had performed 128 cases of thoracoscopic lobectomy in children; the results showed that once meticulous operation was performed, it could effectively avoid common postoperative complications and avoid chest tube placement by a minimally invasive procedure in majority of patients.^[21]^ As a result, the incidence of postoperative complications has reached a low level that was the significant precondition of NCT placement after lobectomy in children. In addition, a report has described that confirmation of consistent pneumostasis at the end of the operation could decrease the probability of postoperative complications and provide an opportunity to omit chest tube drainage after thoracoscopic major lung resection.^[18]^ In our study, we also repeatedly checked and identified operative site to ensure no active bleeding and air leakage before the surgery was completed (Fig. 2). Meanwhile, the advances of operating instruments have been the significant part of the success of minimally invasive surgery. Koga et al showed that using Enseal or Ligasure as the device of choice for sealing lung tissue was safe and efficient and could have a low complication rate. Furthermore, using Enseal to seal the lung tissue may result in a lower complication rate which is more commendable.^[20]^ In our study, we have used both Enseal and Ligasure to seal lung tissue, and the ability of incision and sealing of these 2 instruments was definitely secure.

In this study, we rigorously formulated the standardized criteria shown in Table 1. The patients were selected for the procedure following our standardized criteria for treatment without chest drain after thoracoscopic lobectomy in children. The interlobar fissure disposal is always the conundrum perplexing pediatric thoracic surgeons. Rothenberg and Yamataka et al had reported that in thoracoscopic lobectomy in children, the major obstacle for success is the status of the fissures between lung lobes. Incomplete or absent fissures, which strikingly increases the difficulty of this surgery.^[29,31]^ When we encounter an underdeveloped or not-developed interlobar fissure, the section of the interlobar fissure lung tissue would be larger than other situations; hence, the probability of air leaking and bleeding would accordingly increase. Therefore, we have included this fact in our criteria. Besides, chest infection could increase the difficulty of the surgery and the postoperative complication rate. Yamataka et al also demonstrated that it is much easier to perform thoracoscopic pulmonary lobectomy in cases who have not had chest infection.^[29]^ In this study, we also excluded patients with severe chest cavity infections, which would have the highest risk of intra- and postoperative complications, including pneumothorax, bronchopleural fistula, pleural effusion, bleeding, and residual lesion, as illustrated in a systematic review.^[32]^ If severe extensive adhesions are present in the chest cavity, it would be accompanied with massive bleeding and exudation during adhesiolysis. Kouritas et al demonstrated that patients undergoing major lung resection with pleural adhesions have an increased incidence of adverse surgical outcomes and higher pleural morbidity.^[33]^ Underdeveloped interlobar fissure, severe chest cavity infection or severe adhesions may be contraindications for this procedure; therefore, we have included these in our criteria. This strict selection criteria probably helps patients have a smooth surgery and rehabilitation, and may provide surgeons a safe alternative to the routine use of a postoperative chest drains.

To the best of our knowledge, this is the first report on the absence of chest tube placement after thoracoscopic lobectomy in children, and this study shows that this procedure is safe and effective in the precondition of meticulous resection and strict patient selection. However, this study was retrospective with a small sample size; studies with a larger sample size are needed to confirm its safety and effect.

## Author contributions

**Conceptualization:** Kaisheng Cheng, Miao Yuan, Chang Xu, Gang Yang.

**Data curation:** Kaisheng Cheng.

**Formal analysis:** Kaisheng Cheng, Miao Yuan, Gang Yang.

**Funding acquisition:** Chang Xu, Gang Yang, Min Liu.

**Investigation:** Chang Xu, Gang Yang, Min Liu.

**Methodology:** Kaisheng Cheng, Miao Yuan.

**Project administration:** Chang Xu, Gang Yang, Min Liu.

**Resources:** Kaisheng Cheng, Miao Yuan.

**Software:** Kaisheng Cheng.

**Supervision:** Chang Xu, Gang Yang, Min Liu.

**Validation:** Chang Xu, Min Liu.

**Visualization:** Kaisheng Cheng.

**Writing – original draft:** Kaisheng Cheng.

**Writing – review & editing:** Miao Yuan, Chang Xu.
